# Anterior Cervical Abscess Following Anterior Cervical Discectomy and Fusion Caused by *Moraxella catarrhalis*: A Case Report and Focused Literature Review

**DOI:** 10.3390/jcm15020897

**Published:** 2026-01-22

**Authors:** Helen Mary Hall, Finley Bettsworth, Imran Haq, Mario Ganau

**Affiliations:** 1 Oxford University Medical School, John Radcliffe Hospital, University of Oxford, Oxford OX3 9DU, UK; 2 Department of Neurosurgery, The National Hospital for Neurology and Neurosurgery, London WC1N 3BG, UK; 3 Nuffield Department of Clinical Neurosciences, University of Oxford, Oxford OX3 9DU, UK; 4 School of Medicine, BAU International University Batumi, 237 Fridon Khalvashi ST, 6010 Batumi, Georgia

**Keywords:** anterior cervical discectomy and fusion (ACDF), *Moraxella catarrhalis*, prevertebral abscess, spinal surgery infection, cervical myelopathy, deep surgical site infection, DCM, anterior cervical abscess

## Abstract

**Background:** Anterior cervical discectomy and fusion (ACDF) is widely performed and has a low incidence of postoperative infection. Anterior cervical abscess is a rare but potentially life-threatening complication, typically caused by skin or oral flora. Identification of atypical pathogens has important implications for diagnostic vigilance and antimicrobial management. **Case Presentation:** We report a 56-year-old man with degenerative cervical myelopathy and significant respiratory comorbidity who underwent single-level ACDF and developed progressive dysphagia and neck pain in the early postoperative period. Imaging demonstrated a prevertebral abscess requiring urgent surgical drainage. Intraoperative cultures identified *Moraxella catarrhalis*, a respiratory tract commensal rarely implicated in postoperative spinal infections. No evidence of esophageal perforation or superficial wound contamination was identified. The patient was treated with surgical washout and prolonged culture-directed antibiotic therapy, with full clinical recovery. To contextualize novelty, we performed a focused review of the available literature on *M. catarrhalis* spinal infections. **Conclusions:** This case expands the spectrum of pathogens implicated in postoperative cervical spine infections and highlights the need to consider respiratory tract organisms in high-risk patients, particularly those with chronic pulmonary disease or immunosuppression. Early imaging in the presence of dysphagia, prompt source control, and culture-directed antimicrobial therapy are essential to optimizing outcomes.

## 1. Introduction

Anterior cervical abscesses (ACAs) are a rare but potentially serious surgical complication following anterior cervical discectomy and fusion (ACDF) [1]. ACAs typically present in the early postoperative period with nonspecific symptoms, including neck pain and stiffness, fever, dysphagia, neck swelling or erythema, and neurological deficits [2,3]. Infections following ACDF are most commonly caused by skin and oral flora, with *Staphylococcus aureus* being the most frequently isolated pathogen [1,2]. Other reported organisms include *Streptococcus intermedius*, *Serratia marcescens*, and *Propionibacterium acnes* [4].

Here, we report a case of ACA following ACDF caused by *Moraxella catarrhalis*, an organism that is typically regarded as a benign colonizer of the upper respiratory tract [5]. To the best of our knowledge, *M. catarrhalis* has not previously been reported as the causative pathogen in post-ACDF cervical abscess formation. In this case, abscess development was attributed to transient *M. catarrhalis* bacteremia at the time of surgery. A contributory factor may have been mucosal microtrauma related to endotracheal intubation, highlighting a potentially under-recognized iatrogenic mechanism for postoperative ACA formation.

This case underscores the importance of maintaining a high index of suspicion for atypical pathogens in postoperative spinal infections, particularly in patients with respiratory risk factors such as chronic obstructive pulmonary disease requiring corticosteroid therapy, active smoking, and reduced respiratory reserve secondary to degenerative cervical myelopathy. In addition, it draws attention to the vulnerability of the anterior cervical anatomy in the context of airway management and surgical exposure.

To contextualize the novelty of this case, we performed a focused literature review to situate this report within the existing body of evidence. We therefore present the first reported case of an anterior cervical abscess following ACDF caused by *Moraxella catarrhalis*. This report expands the spectrum of recognized post-ACDF pathogens and emphasizes the need for comprehensive microbiological evaluation and individualized, culture-directed management in postoperative spinal infections.

## 2. Case Presentation

A 56-year-old right-hand-dominant male presented with an 18-month history of progressive cervical myelopathy, characterized by clumsiness, frequent dropping of objects, gait instability, and urinary urgency. His symptoms had deteriorated significantly over the preceding 6 months. His past medical history included chronic obstructive pulmonary disease (COPD), managed with daily oral prednisone (5 mg), and a prior non-ST elevation myocardial infarction (NSTEMI), for which he was taking clopidogrel. He was an active smoker at the time of surgery.

On neurological examination, motor strength was preserved in both upper and lower limbs. However, the patient exhibited bilateral hypoesthesia, reduced proprioception in the lower limbs, brisk reflexes throughout, a positive Babinski reflex, and an unsteady tandem gait. No clonus was observed. Clinical findings proved consistent with advanced DCM. The Modified Japanese Orthopedic Association (mJOA) scale is widely used and validated for assessing the severity of degenerative cervical myelopathy [6]. As per the mJOA scoring system, the patient was found to have a score of 10/18 (indicating severe cervical myelopathy) due to difficulties with fine motor control, mobility aid requirement, sensory disturbance, and bowel and bladder symptoms.

**Figure 1:**
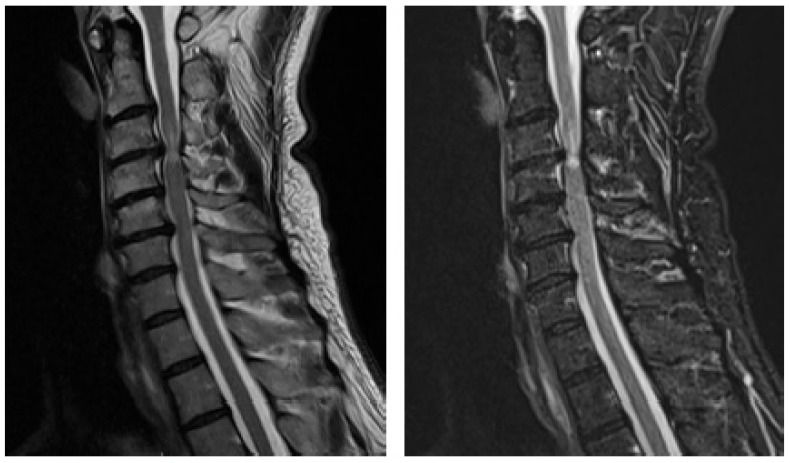
**Pre-operative sagittal T2-weighted MRI of the cervical spine demonstrating severe cervical spinal cord compression at C3–4**. Sagittal T2-weighted MRI of the cervical spine showing degenerative disc disease at the C3–4 level with significant anterior spinal cord compression and associated intramedullary T2 hyperintensity. The increased cord signal is consistent with myelomalacia and correlates with the patient’s progressive neurological deficits, including gait instability, upper limb clumsiness, and sensory disturbance, supporting the indication for surgical decompression.

MRI of the cervical spine demonstrated multilevel degenerative disc disease from C3/C4 to C5/C6, with the most pronounced spinal cord compression and T2-weighted signal hyperintensity at C3/C4, suggestive of myelomalacia (Figure 1).

Following discussion in a multidisciplinary team (MDT) meeting and consultation in the neurosurgical outpatient clinic, the patient was counseled regarding surgical options. These included ACDF at C3/C4 or posterior decompression with laminectomy and fusion from C3 to C6 [7]. After discussion of benefits and risks, including the potential for watchful waiting or a posterior approach with lateral mass screw fixation, the patient consented to proceed with ACDF at C3/C4.

The procedure was performed under general anesthesia without intraoperative complication. Intubation proved difficult as a result of moderate degree of oropharyngeal obstruction (Mallampati Class III) and limited cervical mobility attributed to the patient’s DCM. No mucosal injury was identified intraoperatively.

**Figure 2:**
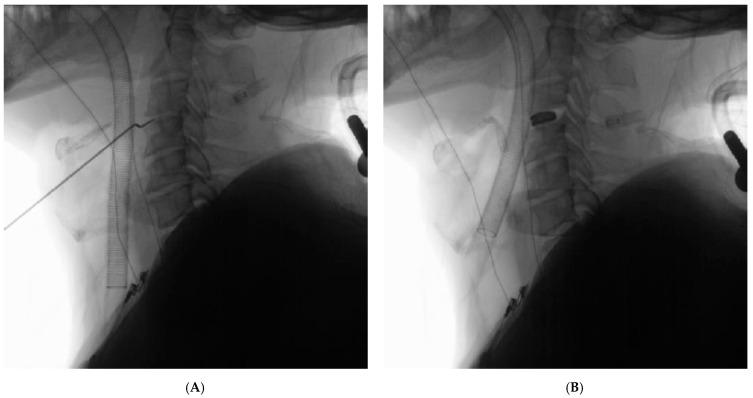
**Intraoperative fluoroscopic lateral radiographs demonstrating C3–4 anterior cervical discectomy and fusion (ACDF)**. (**A**) Intraoperative lateral fluoroscopic radiograph obtained during level localization and alignment assessment prior to discectomy, demonstrating accurate identification of the C3–4 disk space. The endotracheal tube is visible anteriorly, reflecting airway instrumentation in the context of limited cervical mobility. (**B**) Intraoperative lateral fluoroscopic radiograph following placement of the interbody cage, confirming appropriate implant positioning, restoration of disc height, and satisfactory sagittal alignment at the operative level, with no evidence of malposition or instability.

A right-sided anterior cervical approach was used. After disc excision and decompression, a 3D-printed titanium interbody cage with lordotic profile was implanted. No visceral injury nor cerebrospinal fluid (CSF) leak was recorded. Intraoperative fluoroscopy confirmed correct implant placement (Figure 2). Hemostasis was secured, and a subfascial drain was placed prior to layered closure.

The patient was discharged on postoperative day-1 in a stable condition without any clinical concerns. This early discharge was in line with enhanced recovery after surgery (ERAS) principles, aiming to reduce hospital stays and complications while promoting early mobilization [8]. By day 5, he developed mild dysphagia and low-grade fever. Clinical examination was unremarkable, and laboratory findings revealed normal leukocyte count with mildly elevated C-reactive protein (CRP) and erythrocyte sedimentation rate (ESR). A cervical CT scan was obtained but showed stable positioning of the C3–4 interbody cage without evidence of hardware migration, subsidence, or prevertebral collection. The patient was discharged with advice for symptomatic care.

Two days later (postoperative day 7), the patient re-presented with worsening neck pain, swelling, and tenderness. Repeat bloods demonstrated stable inflammatory markers. Given clinical suspicion for deep space infection, the patient underwent a diagnostic workup, starting with dynamic barium swallow studies. Lateral barium swallow fluoroscopy demonstrated mild prevertebral impression without evidence of contrast leak, and anteroposterior view confirmed symmetrical esophageal transit.

**Figure 3:**
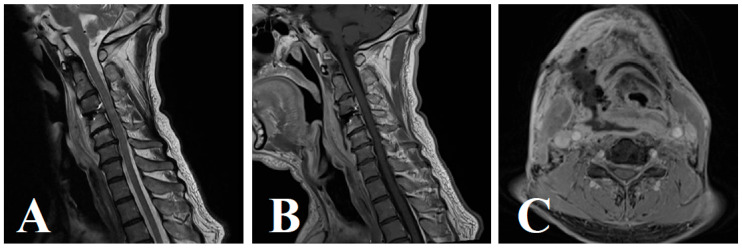
**Postoperative imaging on day 7 evaluating worsening neck pain and dysphagia following C3–4 ACDF**. (**A**) Sagittal T2-weighted MRI demonstrating a well-defined hyperintense prevertebral fluid collection anterior to the C3–4 vertebral bodies, resulting in posterior displacement of the pharynx and esophagus. This mass effect explains the patient’s progressive dysphagia. (**B**) Sagittal post-contrast T1-weighted MRI showing rim enhancement of the prevertebral collection, consistent with an organized abscess exerting anterior-to-posterior compression on the aerodigestive tract. (**C**) Axial post-contrast T1-weighted MRI confirming lateral displacement and compression of the esophagus by the enhancing collection, correlating with the patient’s swallowing difficulty and local neck discomfort.

Given the persistent symptoms, an urgent contrast-enhanced MRI of the cervical spine was obtained. Imaging revealed a rim-enhancing prevertebral fluid collection at the C3–4 level, with displacement of the trachea and esophagus, consistent with a deep cervical abscess (Figure 3A–C). These imaging studies confirmed the presence of a prevertebral abscess, prompting urgent surgical exploration and drainage.

The patient underwent emergent surgical exploration and drainage of the prevertebral abscess. Approximately 30 mL of purulent material was evacuated. ENT consultation confirmed no evidence of esophageal perforation or other visceral injury. Intraoperative ultrasound (IoUS) confirmed complete washout of the surgical site. Intraoperative cultures identified *Moraxella catarrhalis*, a respiratory commensal rarely implicated in postoperative spinal infections. Intraoperative swabs from the skin and suprafascial layers were also obtained and yielded no bacterial growth. In addition, extended cultures of deep neck wound specimens showed no growth of *Actinomyces* species after 2 weeks of incubation.

The organism was resistant to amoxicillin but sensitive to trimethoprim–sulfamethoxazole and azithromycin. In consultation with the infectious diseases team, the patient was commenced on a 3-month course of dual oral antibiotics (trimethoprim-sulfamethoxazole [TMP-SMX] and azithromycin) guided by microbiological sensitivities and clinical response. No further surgical intervention was required.

All microbiological analyses were performed by the hospital diagnostic microbiology laboratory as part of routine clinical care; representative intraoperative microscopy or culture plate images were therefore not available for inclusion, and results are described in the text.

**Figure 4:**
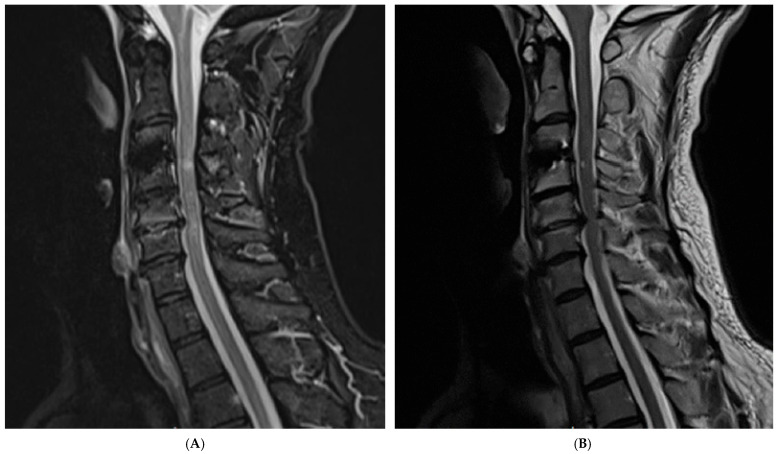
**6-month postoperative MR images demonstrating resolution of infection and stable fusion**. (**A**) Sagittal T2-weighted MRI showing complete resolution of prevertebral soft-tissue edema and absence of residual fluid collection, with normalization of surrounding anatomical structures. (**B**) Sagittal T1-weighted MRI confirming maintained position of the interbody implant without abnormal enhancement or evidence of recurrent abscess formation. These findings correlate with the patient’s clinical recovery, resolution of dysphagia, and return to neurological baseline.

At 6-month follow-up, the patient demonstrated marked clinical improvement, with resolution of dysphagia and return to neurological baseline. Repeat MRI showed no residual abscess, spinal cord compression, or new instability (Figure 4). No recurrent infection was observed, no long-term antibiotic-related adverse effects were reported, and the fusion construct remained well-aligned.

**Figure 5:**
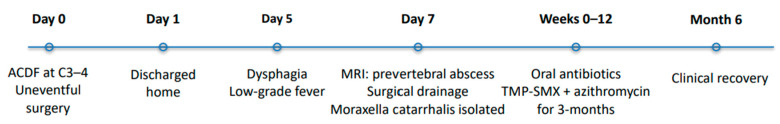
**Timeline of clinical course**. Timeline summarizing the patient’s perioperative course, symptom onset, diagnostic imaging, surgical reintervention, antimicrobial therapy, and clinical outcome following anterior cervical discectomy and fusion. TMP-SMX = trimethoprim–sulfamethoxazole.

Detailed perioperative timelines and laboratory values are available from the authors on request. The clinical course is summarized in Figure 5.

## 3. Discussion and Review of the Literature

ACDF is the most commonly adopted anterior approach to surgically address cervical spine pathologies [1,9], with alternative surgical corridors being the lateral and posterior routes [7,10]. Our patient underwent ACDF, having presented with symptoms associated with cervical myelopathy including clumsiness, urinary symptoms, unsteady gait, and sensory disturbance. Following mJOA scoring, this was found to be a case of severe cervical myelopathy with an mJOA score of 10/18 [6]. Following the patient’s ACDF, he re-presented on postoperative day 5 with mild dysphagia and low-grade fever, which was later found to be an ACA, a form of deep surgical site infection. Surgical drainage and intraoperative cultures revealed the causative agent to be *M. catarrhalis*, a benign colonizer of the upper respiratory tract [5]. This case highlights how uncommon respiratory tract organisms may cause serious postoperative spinal infections in selected high-risk patients, despite adherence to standard perioperative protocols.

### 3.1. Pathogen Characteristics

To the best of our knowledge, the current literature has no reported cases of ACA formation following ACDF attributed to *M. catarrhalis*. Rather, infections following ACDF are most commonly caused by skin and oral flora, with *Staphylococcus aureus* being the most frequently isolated pathogen [1,2]. The typical microorganisms involved in post-ACDF anterior cervical deep wound infections also include *Streptococcus* species and anaerobic bacteria [1,2,5]. Indeed, several causative pathogens have been implicated in both early and late infectious complications following spinal surgery. However, the vast majority of reported organisms are derived from cutaneous or oropharyngeal flora, making the identification of *M. catarrhalis* in this context particularly unusual.

In contrast to typical post-ACDF pathogens such as *Staphylococcus aureus*, streptococcal species, and anaerobic flora, *Moraxella catarrhalis* is primarily a respiratory tract commensal [5]. This distinction is clinically relevant because empiric regimens for suspected postoperative cervical infections often prioritize coverage of skin and oral flora, with subsequent narrowing based on culture results [4]. Identification of a respiratory commensal in a deep surgical site should prompt consideration of host factors and perioperative events that may predispose to transient bacteremia, particularly in patients with chronic pulmonary disease or immunosuppression.

The emergence of *M. catarrhalis* as a causative agent in a post-ACDF ACA is unprecedented, highlighting the evolving microbial landscape in the context of spinal surgery infectious complications. We have identified reports of spinal infections attributed to *Moraxella*, although none occurred in the context of cervical spine surgery, and none described prevertebral or anterior cervical abscess formation following ACDF. To contextualize the novelty of this case, we performed a focused narrative review of the published literature on spinal infections attributable to *Moraxella catarrhalis*. All cases of *M. catarrhalis* spinal infection meeting inclusion criteria are documented in Table 1.

A review of the published literature identified four non-retracted reports of spinal infection attributable to *Moraxella catarrhalis*. These comprised two cases of vertebral osteomyelitis (Prallet et al.; Mousa et al.), one case of concomitant discitis and bacteremia (Brunckhorst et al.), and one case of vertebral osteomyelitis associated with infective endocarditis (Maierean et al.). All reported cases were attributed to hematogenous seeding, most frequently in medically vulnerable hosts with chronic respiratory disease or immunosuppression. None occurred in the context of cervical spine surgery, and none described prevertebral or anterior cervical abscess formation. To our knowledge, no previous postoperative spinal infections attributable to *Moraxella catarrhalis* have been reported.

### 3.2. Mechanism of Infection

Given that patients with chronic obstructive pulmonary disease (COPD) are frequently colonized with *Moraxella catarrhalis* and are predisposed to transient bacteremia, hematogenous dissemination represents a compelling explanation for the present case [15,16,17]. However, the localized prevertebral distribution of the infection, its delayed postoperative onset, and the organism’s origin as an upper airway commensal also make airway instrumentation with transient mucosal microtrauma a plausible contributory mechanism [5]. It is therefore likely that a combination of heavy airway colonization and perioperative vulnerability facilitated pathogen entry, culminating in the first described anterior cervical abscess following ACDF caused by *M. catarrhalis*.

Additional microbiological findings further support a hematogenous rather than iatrogenic mechanism of infection. Preoperative nasal screening for methicillin-resistant *Staphylococcus aureus* (MRSA) was negative, and intraoperative swabs obtained from the skin and suprafascial layers at the time of surgical washout demonstrated no microbial growth. In addition, prolonged culture of deep neck wound specimens showed no growth of *Actinomyces* species after two weeks of incubation, arguing against preexisting colonization, superficial wound contamination, or a chronic cervicofacial or contiguous oropharyngeal source of infection. In contrast, the causative organism isolated from the deep surgical site is a recognized respiratory tract commensal, supporting transient bacteremia as the most likely mechanism of dissemination, despite the absence of identifiable aerodigestive tract injury. Importantly, transient bacteremia may resolve rapidly, meaning that negative blood cultures at re-presentation do not exclude a hematogenous source.

These observations are consistent with previous reports suggesting that *M. catarrhalis* may act as an opportunistic pathogen in vulnerable hosts [11,12,13,14]. Maierean et al. described a patient with COPD who developed vertebral osteomyelitis attributed to *M. catarrhalis*, presumed to have arisen via hematogenous spread, albeit in a non-postoperative setting [11]. Given the vulnerable local anatomy involved in anterior cervical spinal surgery, this pathogenic mechanism may pose an even greater risk for infectious complications in patients with similar respiratory and immunological risk profiles.

Taken together, these findings support the concept that *M. catarrhalis* may act as an opportunistic pathogen when host defenses are impaired, even in the absence of overt mucosal disruption.

### 3.3. Perioperative Considerations in High-Risk Patients

Patients receiving long-term corticosteroid therapy or those with chronic respiratory disease represent a higher-risk subgroup for postoperative infection following ACDF [1,4]. Preoperative optimization should include careful respiratory assessment, smoking cessation counseling, and multidisciplinary perioperative planning involving anesthesia, infectious diseases, and respiratory medicine [8]. Although routine extended postoperative antibiotic prophylaxis is not currently recommended following clean spinal surgery [18,19,20], heightened vigilance is warranted in immunosuppressed patients. In such cases, early postoperative review, a low threshold for imaging in the presence of dysphagia or neck symptoms, and prompt culture-directed antimicrobial therapy are essential. At present, evidence does not support routine prolonged prophylactic antibiotics solely based on corticosteroid use; however, individualized risk assessment is advised [4,21]. These considerations may be particularly relevant as ACDF is increasingly performed in older patients with complex medical comorbidities.

### 3.4. Timelines for Infection

The rate of occurrence of postoperative infections following ACDF is cited as 0.07% (range 0–0.39%) [22], indicating that ACDF is associated with a low overall risk of postoperative infection. Among these, ACAs are uncommon complications. Risk is increased in medically vulnerable patients and in the setting of instrumentation; longer operative time is also associated with increased adverse events after ACDF [21,23,24]. In the case of our patient, his key risk factors for infection included his steroid-controlled COPD, resulting in reduced immune capacity, as well as the COPD itself, with point prevalence studies indicating that 5 to 32% of adults with COPD are colonized with *M. catarrhalis* at any given time, making it a particularly common commensal bacteria in this demographic [15,16,17].

Most postoperative infections occur in the early postoperative period; however, delayed deep infections have been reported months to years after ACDF [2,25,26]. Postoperative infection may result from intraoperative inoculation, hematogenous seeding, or less commonly, contiguous spread from adjacent structures [5,23]. We postulate that given the lack of intraoperative damage to the aerodigestive viscera, the development of our patient’s ACA can be attributed to an *M. catarrhalis* bacteremia present at the time of surgery, made likely by his aforementioned pulmonary risk factors. This temporal pattern is consistent with early hematogenous seeding rather than delayed contiguous spread.

Delayed presentations (>6 months) warrant careful evaluation for occult aerodigestive pathology or delayed esophageal perforation in the context of anterior cervical instrumentation. Christiano et al. reported a prevertebral abscess presenting with acute dysphagia 2 years after ACDF without an identifiable mucosal breach at exploration [26]. Similarly, Jin et al. described infection presenting with dysphagia 20 years after ACDF, again without clear predisposing features at the index procedure [25]. In our patient, intubation was difficult (Mallampati class III), and although no visceral injury was identified intraoperatively, minor mucosal microtrauma cannot be excluded. Nevertheless, the overall findings support a hematogenous route as the most likely mechanism of bacterial translocation.

### 3.5. Treatment Strategy and Outcomes

Understanding the pathogenesis in our patient allows us to contextualize current best practices in the management of anterior cervical abscesses following ACDF. Management of ACAs following ACDF typically involves a combination of surgical treatment and systemic antibiotic therapy. Recent multicenter experience with minimally invasive surgical management of cervical spondylodiscitis further supports the importance of early source control and multidisciplinary decision-making in deep cervical infections, with surgical strategy tailored to anatomy, stability, and patient comorbidity [27]. Following surgical intervention, patients are usually initiated on empirical broad-spectrum IV antibiotics, such as Vancomycin for MRSA cover in combination with a 3rd generation Cephalosporin such as Ceftriaxone or Cefepime to cover Gram-negative pathogens [19,21,24]. Once culture and sensitivity results are available, antibiotic therapy should be narrowed accordingly. Our case suggests these principles may still be applied, though vigilance is needed when culture reveals atypical pathogens.

A defining microbiological characteristic of *M. catarrhalis* is its ability to produce BRO-type beta-lactamases, which confer resistance to penicillin and amoxicillin in the majority of clinical isolates [5,28]. This resistance profile necessitates careful antimicrobial selection and highlights the importance of culture-directed therapy in invasive infections. Agents such as third-generation cephalosporins, macrolides, fluoroquinolones, and trimethoprim–sulfamethoxazole generally retain activity against this organism, though resistance patterns may vary regionally [5,28].

The typical duration of antibiotic therapy for deep postoperative spinal infections ranges from 6 to 8 weeks, beginning with IV administration and, in cases with good clinical and laboratory response, transitioning to an oral step-down regimen [19,20]. Monitoring of CRP and ESR is essential during therapy to guide response and duration [21]. In selected patients with effective source control and favorable clinical and laboratory response, an early transition to oral therapy may be appropriate, although regimens and duration should be individualized in conjunction with infectious diseases specialists [19,21,24]. Prolonged therapy and close follow-up are particularly important in the setting of spinal instrumentation [29], where incomplete treatment may increase the risk of recurrence or persistent infection [23,30].

In this case, prompt surgical source control combined with prolonged culture-directed antimicrobial therapy resulted in complete clinical and radiological resolution without recurrence.

### 3.6. Antibiotic Management of Atypical Pathogens

Antibiotic therapy remains the cornerstone of treatment for invasive deep surgical site infections, including those attributable to *Moraxella catarrhalis*. Antimicrobial management should be guided by culture and susceptibility results whenever possible. In a previously reported case of vertebral osteomyelitis caused by *M. catarrhalis*, initial treatment with intravenous ceftriaxone followed by oral doxycycline was effective [11]. In general, antibiotic therapy for spinal infections is administered over several weeks, with duration and regimen adjusted according to clinical response and inflammatory markers [19,20,21]. In the postoperative setting, surgical debridement combined with prolonged antimicrobial therapy remains the mainstay of management [24], particularly in the presence of compressive symptoms, neurological deficit, or failure of conservative treatment [19,21,24].

The emergence of *M. catarrhalis* in invasive spinal infections also necessitates consideration of its characteristic antimicrobial resistance patterns. Most clinical isolates produce beta-lactamases, which may confer resistance to penicillin and amoxicillin [5,28]. In the present case, empirical antibiotic therapy was initiated promptly and subsequently refined based on microbiological sensitivities, underscoring the importance of culture-directed treatment in atypical postoperative infections.

### 3.7. Implications for Airway Management and Surgical Protocols

Given the widespread use of ACDF and continued advances in spinal instrumentation and perioperative care [1], clinicians must remain vigilant for rare and atypical pathogens that may be introduced through respiratory manipulation, such as endotracheal intubation. This is particularly relevant when patients present with unexpected organisms or delayed postoperative symptoms. Early multidisciplinary involvement, including consultation with infectious diseases specialists and meticulous microbiological evaluation, is critical to optimizing outcomes in these unconventional presentations [19,21,24]. These considerations should be integrated with patient-specific risk stratification, particularly in populations known to be at increased risk of postoperative infection [4,21,24].

In the present case, dynamic barium swallow studies demonstrated no evidence of esophageal or tracheal injury and no fistulous communication, making direct iatrogenic contamination of the surgical site unlikely and supporting a hematogenous mechanism of infection. This interpretation is reinforced by the absence of superficial wound contamination and negative cultures from the skin and suprafascial layers.

This mechanism is particularly plausible in the context of this patient’s multiple risk factors for transient bacteremia, including chronic obstructive pulmonary disease, long-term oral corticosteroid use, active smoking, reduced cervical mobility due to degenerative cervical myelopathy, and upper airway manipulation during intubation. Although *M. catarrhalis* is rarely reported as a cause of postoperative spinal infection, existing reports of invasive disease most commonly support hematogenous seeding in medically vulnerable hosts [11,12,13,14]. In the perioperative setting, transient mucosal microtrauma related to airway instrumentation may plausibly facilitate bacteremia, although this mechanism is difficult to confirm in individual cases.

Overall, this case reinforces the need for heightened postoperative surveillance in high-risk spinal surgery patients and consideration of respiratory tract commensals as potential causative pathogens. Importantly, negative blood cultures at the time of clinical re-presentation do not exclude a transient perioperative bacteremia, as bacteremia may have resolved or been suppressed by prior antimicrobial exposure, limiting diagnostic yield.

As ACDF continues to be widely performed, clinicians should remain alert to rare but clinically significant postoperative complications [1]. The identification of *Moraxella catarrhalis* as the causative organism in an anterior cervical abscess following ACDF expands the recognized spectrum of postoperative spinal pathogens and highlights the importance of comprehensive microbiological evaluation and tailored antimicrobial therapy [19,21,24]. In patients with pulmonary comorbidities, particularly COPD and chronic corticosteroid exposure, respiratory tract organisms such as *M. catarrhalis* may warrant increased consideration in the differential diagnosis of postoperative deep cervical infection [5,15,16,17].

Given the limited number of reported cases, specific management guidelines for postoperative spinal infection caused by *M. catarrhalis* are lacking. Accordingly, treatment should follow established principles for deep postoperative spinal infection, including timely surgical source control when indicated and antimicrobial therapy tailored to susceptibility results [11,12,13,14,19,21,24]. Careful evaluation for aerodigestive injury remains essential in patients presenting with postoperative dysphagia, and adjunctive intraoperative tools, such as ultrasound, may assist in confirming adequate surgical washout in selected cases. Finally, this case highlights the value of early multidisciplinary collaboration between spine surgery, otolaryngology, and infectious diseases in optimizing outcomes for complex postoperative infections [19,21,24].

## 4. Conclusions

Anterior cervical abscess formation following anterior cervical discectomy and fusion is rare, but when it occurs, it carries significant morbidity and requires prompt recognition and management. This case demonstrates that postoperative deep cervical infection may arise from atypical organisms not traditionally associated with spinal surgery, including respiratory tract commensals such as *Moraxella catarrhalis*.

The identification of this pathogen in a prevertebral abscess following ACDF highlights the importance of maintaining diagnostic vigilance in medically vulnerable patients, particularly those with chronic pulmonary disease, smoking history, or long-term corticosteroid exposure. In such individuals, early postoperative symptoms such as dysphagia or neck discomfort should prompt a low threshold for advanced imaging, even in the absence of marked laboratory abnormalities.

Effective management relies on timely surgical source control, thorough microbiological evaluation, and tailored antimicrobial therapy based on susceptibility testing. This case expands the spectrum of recognized pathogens in postoperative cervical spine infection and underscores the need for individualized, multidisciplinary care when managing uncommon but clinically significant complications of ACDF.

## Figures and Tables

**Table 1:** Summary of reported spinal infections caused by *Moraxella catarrhalis*, including the present case.

Reference	Patient Description	Risk Factors	Surgical Context (Yes [Y], No [N])	Treatment
Maierean et al., 2019 [11]	65-year-old female developed vertebral osteomyelitis due to *M. catarrhalis*; presumed hematogenous spread.	Immunosuppression (Azathioprine treatment for Crohn’s disease).	N	IV ceftriaxone for 8 weeks, followed by oral doxycycline for 3 months.
Brunckhorst et al., 2020 [12]	70-year-old male developed T3/4 discitis with *M. catarrhalis* positive blood cultures.	Elderly.	N	4-week ceftriaxone followed by 1 g ampicillin PO TDS for 2 weeks
Mousa, 2003 [13]	52-year-old male developed a gluteal abscess. Aspirate culture revealed *Mycobacterium tuberculosis* and *M. catarrhalis*.	Nil stated.	N	IV ampiclox (duration not stated). Surgical drainage. Anti-TB chemotherapy for 2 months.
Prallet et al., 1991 [14]	68-year-old male developed spontaneous lumbar pain over 12 months. Diagnosed with vertebral osteomyelitis. Aspirate cultures revealed *M. catarrhalis*.	Long-term tobacco use.	N	IV cefmenoxime and pefloxacin for 6 weeks, followed by PO pefloxacin and pristinamycin for 10 weeks.
Current case (present study)	56-year-old male with prevertebral abscess following C3-4 ACDF. *M. catarrhalis* isolated.	Smoking history, COPD, corticosteroid treatment	Y	Surgical washout, 3-month course of dual oral antibiotics (trimethoprim-sulfamethoxazole and azithromycin)

## Data Availability

The data presented in this study are available on request from the corresponding author due to patient confidentiality.

## Abbreviations

The following abbreviations are used in this manuscript:

ACA	Anterior cervical abscess
ACDF	Anterior cervical discectomy and fusion
COPD	Chronic obstructive pulmonary disease
CRP	C-reactive protein
CT	Computed Tomography
DCM	Degenerative cervical myelopathy
ENT	Ear Nose and Throat
ERAS	Enhanced Recovery after Surgery
ESR	Erythrocyte sedimentation rate
HIV	Human immunodeficiency virus
IoUS	Intraoperative ultrasound
MDT	Multidisciplinary Team
mJOA	Modified Japanese Orthopedic Association
MRI	Magnetic Resonance Imaging
MRSA	Methicillin-Resistant Staphylococcus aureus
NSTEMI	Non-ST elevation myocardial infarction
PO	Per os
TB	Tuberculosis
TMP-SMX	Trimethoprim–sulfamethoxazole
